# A comprehensive update of the sequence and structure classification of kinases

**DOI:** 10.1186/1472-6807-5-6

**Published:** 2005-03-16

**Authors:** Sara Cheek, Krzysztof Ginalski, Hong Zhang, Nick V Grishin

**Affiliations:** 1 Howard Hughes Medical Institute, University of Texas Southwestern Medical Center 5323 Harry Hines Blvd., Dallas, Texas 75390, USA; 2 Department of Biochemistry, University of Texas Southwestern Medical Center 5323 Harry Hines Blvd., Dallas, Texas 75390, USA; 3 Bioinformatics Laboratory, Interdisciplinary Centre for Mathematical and Computational Modelling Warsaw University, Pawinskiego 5a, 02-106 Warsaw, Poland

## Abstract

**Background:**

A comprehensive update of the classification of all available kinases was carried out. This survey presents a complete global picture of this large functional class of proteins and confirms the soundness of our initial kinase classification scheme.

**Results:**

The new survey found the total number of kinase sequences in the protein database has increased more than three-fold (from 17,310 to 59,402), and the number of determined kinase structures increased two-fold (from 359 to 702) in the past three years. However, the framework of the original two-tier classification scheme (in families and fold groups) remains sufficient to describe all available kinases. Overall, the kinase sequences were classified into 25 families of homologous proteins, wherein 22 families (~98.8% of all sequences) for which three-dimensional structures are known fall into 10 fold groups. These fold groups not only include some of the most widely spread proteins folds, such as the Rossmann-like fold, ferredoxin-like fold, TIM-barrel fold, and antiparallel β-barrel fold, but also all major classes (all α, all β, α+β, α/β) of protein structures. Fold predictions are made for remaining kinase families without a close homolog with solved structure. We also highlight two novel kinase structural folds, riboflavin kinase and dihydroxyacetone kinase, which have recently been characterized. Two protein families previously annotated as kinases are removed from the classification based on new experimental data.

**Conclusion:**

Structural annotations of all kinase families are now revealed, including fold descriptions for all globular kinases, making this the first large functional class of proteins with a comprehensive structural annotation. Potential uses for this classification include deduction of protein function, structural fold, or enzymatic mechanism of poorly studied or newly discovered kinases based on proteins in the same family.

## Background

We restrict the definition of "kinase" to enzymes that catalyze the transfer of the terminal phosphate group from ATP (with a few exceptions, such GTP, as discussed below) to a substrate containing an alcohol, nitrogenous, carboxyl, or phosphate group as the phosphoryl acceptor. The substrate of a kinase can be a small molecule, lipid, or protein. Kinases play indispensable roles in numerous cellular metabolic and signalling pathways, and they are among the best-studied enzymes at the structural, biochemical, and cellular levels. Despite that all kinases use the same phosphate donor (in most cases, ATP) and catalyze apparently the same phosphoryl transfer reaction, they display remarkable diversity in their structural folds and substrate recognition mechanisms. This is probably due largely to the extraordinarily diverse nature of the structures and properties of their substrates.

In order to investigate the relationship between structural fold and functional specificities in kinases, we carried out a comprehensive analysis of all available kinase structures and sequences [1] three years ago. This analysis surveyed more than 17,000 kinase sequences, which were classified into 30 families of homologous proteins. Furthermore, we found that 98% of these sequences fell into seven general fold groups with known structures. We were subsequently able to use this kinase classification scheme to analyze various aspects of kinase function and evolution, such as the shared catalytic and nucleotide substrate binding mechanisms across different kinase families and folds.

Such protein classification has been shown to be in demand by biologists because it is a useful tool for analyzing various aspects of sequence/structure/function relationships in proteins, such as structure prediction or identification of functionally important residues. Given the rapid increase in the sizes of sequence and structure databases, it is also essential that a protein classification scheme remain stable over time. Ideally, the backbone of a classification scheme should not require fundamental revisions with the inclusion of additional information. The nearly three years that have passed since the completion of the initial kinase survey have seen a large influx of sequence and structural data due to large-scale projects such as genome sequencing and structural genomics initiatives, as well as functional studies by numerous individual research laboratories. There appear to be sufficient new developments in the field that warrant an update of the initial kinase survey and an evaluation of whether the families and fold groups identified in the original classification scheme still provide a comprehensive framework for all available kinase sequences.

Here, we present an updated version of this kinase classification. This update serves two important purposes: to validate the robustness of the initial kinase classification scheme and to present, for the first time, a complete structural annotation of this large functional class of proteins. The updated kinase survey now includes over 59,000 sequences that belong to 25 families of homologous proteins. Despite that the total number of kinase sequences has increased more than three-fold, the framework of the original classification remains sufficient for describing all available kinase sequences. The initial survey also included large-scale structure predictions for kinases with unknown structure. The structures of several of these kinases have now been solved and the original fold group/family placements are shown to be correct. The two new kinase folds that have been characterized recently are discussed. Fold predictions were performed for those kinase families still lacking a homolog with solved structure.

## Results and discussion

Overall, 59,402 sequences are classified into 25 families of homologous kinases. These kinase families are further assembled into 12 fold groups based on similarity of structural fold. 22 of the 25 families belong to 10 fold groups for which the structural fold is known. One additional family (polyphosphate kinase) is now associated with a predicted structural fold and presently forms a distinct fold group. The two remaining families are both integral membrane kinases and comprise the final fold group. Within a fold group, the core of the nucleotide-binding domain of each family has the same architecture, and the topology of the protein core is either identical or related by circular permutation. Homology between families within a fold group is not implied.

The updated kinase classification is summarized in Tables 1 and 2. For each fold group and family therein, the Pfam/COG members are listed as well as the specific kinase activities and a corresponding representative PDB or gi (NCBI gene identification) accession number. The total number of sequences in each family and fold group is also specified. The EC (Enzyme Commission) activities in bold have solved structures. Asterisks indicate that the first structure associated with that kinase activity was solved after the initial kinase classification was completed. Underlined entries are new kinase activities that were not included in the previous classification, although activities added due to re-organization or updating of the EC database are not underlined. It should be noted that the activity lists are not exhaustive, as they include only those kinase activities that have been annotated so far. Of the 190 kinases listed in the EC system over our chosen range (EC2.7.1.- through EC2.7.4.-), 126 activities can be placed in our kinase families. Enzymes that do not use ATP as the phosphate donor are intentionally excluded, except in cases where such kinases fall under existing families (for example, 2.7.1.90: diphosphate – fructose-6-phosphate 1-phosphotransferase in the phosphofructokinase-like family or 2.7.1.147: ADP-dependent glucokinase in the ribokinase-like family). Sequences for the remaining kinase activities have not been identified and thus are not included in our analysis, although it is possible that some of the unannotated kinases may be among the sequences with only general kinase function annotation, such as "similar to such-and-such kinase".

**Table 1:** Classification of kinase activities by family and fold group, part 1.

*Group*	*Family and PFAM/COG members*	*Kinase Activity (E.C.)*	*Representative PDB or gi*
Group 1: protein S/T-Y kinase/ atypical protein kinase/ lipid kinase/ ATP-grasp *23124 sequences*	protein S/T-Y kinase/ atypical protein kinase: COG0478, COG2112, PF00069, PF00454, PF01163, PF01633 *22074 sequences*	2.7.1.32 Choline kinase (*)	PDB: 1cdk
		**2.7.1.37 Protein kinase**	
		**2.7.1.38 Phosphorylase kinase**	
		2.7.1.39 Homoserine kinase	
		2.7.1.67 1-phosphatidylinositol 4-kinase	
		2.7.1.72 Streptomycin 6-kinase	
		2.7.1.82 Ethanolamine kinase	
		2.7.1.87 Streptomycin 3"-kinase	
		**2.7.1.95 Kanamycin kinase**	
		2.7.1.100 5-methylthioribose kinase	
		2.7.1.103 Viomycin kinase	
		2.7.1.109 [Hydroxymethylglutaryl-CoA reductase (NADPH_2_)] kinase	
		**2.7.1.112 Protein-tyrosine kinase**	
		2.7.1.116 [Isocitrate dehydrogenase (NADP+)] kinase	
		**2.7.1.117 [Myosin light-chain] kinase**	
		2.7.1.119 Hygromycin-B kinase	
		**2.7.1.123 Calcium/calmodulin-dependent protein kinase**	
		2.7.1.125 Rhodopsin kinase	
		2.7.1.126 [Beta-adrenergic-receptor] kinase (*)	
		2.7.1.129 [Myosin heavy-chain] kinase	
		2.7.1.135 [Tau protein] kinase (*)	
		2.7.1.136 Macrolide 2'-kinase	
		**2.7.1.137 1-phosphatidylinositol 3-kinase**	
		2.7.1.141 [RNA-polymerase]-subunit kinase	
		2.7.1.153 Phosphatidylinositol-4,5-bisphosphate 3-kinase	
		2.7.1.154 Phosphatidylinositol-4-phosphate 3-kinase	
	
	lipid kinase: PF01504 *321 sequences*	**2.7.1.68 1-phosphatidylinositol-4-phosphate 5-kinase**	PDB: 1bo1
		2.7.1.127 1D-myo-inositol-trisphosphate 3-kinase (*)	
		2.7.1.140 Inositol-tetrakisphosphate 5-kinase	
		2.7.1.149 1-phosphatidylinositol 5-phosphate 4-kinase	
		2.7.1.150 1-phosphatidylinositol 3-phosphate 5-kinase	
		2.7.1.151 Inositol-polyphosphate multikinase	
		2.7.4.21 Inositol-hexakisphosphate kinase	
	
	ATP-grasp: PF01326 *729 sequences*	2.7.1.134 Inositol-tetrakisphosphate 1-kinase	PDB: 1dik
		**2.7.9.1 Pyruvate, phosphate dikinase**	
		2.7.9.2 Pyruvate, water dikinase	

Group 2: Rossmann-like *17071 sequences*	P-loop kinases: COG0645, COG1618, COG1663, COG1936, COG2019, PF00265, PF00406, PF00485, PF00625, PF00693, PF01121, PF01202, PF01583, PF01591, PF01712, PF02223, PF02224, PF02283 *7732 sequences*	2.7.1.12 Gluconokinase (*)	PDB: 1qf9
		**2.7.1.19 Phosphoribulokinase**	
		**2.7.1.21 Thymidine kinase**	
		**2.7.1.22 Ribosylnicotinamide kinase**	
		**2.7.1.24 Dephospho-CoA kinase**	
		**2.7.1.25 Adenylylsulfate kinase**	
		**2.7.1.33 Pantothenate kinase**	
		2.7.1.37 Protein kinase (bacterial)	
		2.7.1.48 Uridine kinase (*)	
		**2.7.1.71 Shikimate kinase**	
		2.7.1.74 Deoxycytidine kinase (*)	
		2.7.1.76 Deoxyadenosine kinase	
		2.7.1.78 Polynucleotide 5'-hydroxyl-kinase (*)	
		**2.7.1.105 6-phosphofructo-2-kinase**	
		**2.7.1.113 Deoxyguanosine kinase**	
		2.7.1.130 Tetraacyldisaccharide 4'-kinase	
		2.7.1.145 Deoxynucleoside kinase (*)	
		**2.7.1.156 Adenosylcobinamide kinase**	
		2.7.4.1 Polyphosphate kinase	
		2.7.4.2 Phosphomevalonate kinase	
		**2.7.4.3 Adenylate kinase**	
		2.7.4.4 Nucleoside-phosphate kinase	
		**2.7.4.8 Guanylate kinase**	
		**2.7.4.9 Thymidylate kinase**	
		**2.7.4.10 Nucleoside-triphosphate – adenylate kinase**	
		**2.7.4.13 (Deoxy)nucleoside-phosphate kinase**	
		**2.7.4.14 Cytidylate kinase**	
		**2.7.4.- Uridylate kinase**	
	
	phosphoenolpyruvate carboxykinase: COG1493, PF01293, PF00821 *815 sequences*	**2.7.1.37 Protein kinase (HPr kinase/phosphatase)**	PDB: 1aq2
		**4.1.1.32 Phosphoenolpyruvate carboxykinase (GTP)**	
		**4.1.1.49 Phosphoenolpyruvate carboxykinase (ATP)**	
	
	phosphoglycerate kinase: PF00162 *1351 sequences*	**2.7.2.3 Phosphoglycerate kinase**	PDB: 13pk
		2.7.2.10 Phosphoglycerate kinase (GTP)	
	
	aspartokinase: PF00696 *2171 sequences*	**2.7.2.2 Carbamate kinase**	PDB: 1b7b
		2.7.2.4 Aspartate kinase	
		2.7.2.8 Acetylglutamate kinase (*)	
		2.7.2.11 Glutamate 5-kinase	
		2.7.4.- Uridylate kinase	
	
	phosphofructokinase-like: PF00365, PF00781, PF01219, PF01513 *1998 sequences*	**2.7.1.11 6-phosphofructokinase**	PDB: 4pfk
		2.7.1.23 NAD(+) kinase (*)	
		2.7.1.56 1-phosphofructokinase	
		2.7.1.90 Diphosphate – fructose-6-phosphate 1- phosphotransferase (*)	
		2.7.1.91 Sphinganine kinase	
		2.7.1.107 Diacylglycerol kinase	
		2.7.1.138 Ceramide kinase	
	
	ribokinase-like: PF00294, PF01256, PF02110 *2722 sequences*	**2.7.1.2 Glucokinase**	PDB: 1rkd
		2.7.1.3 Ketohexokinase	
		2.7.1.4 Fructokinase	
		2.7.1.11 6-phosphofructokinase	
		**2.7.1.15 Ribokinase**	
		**2.7.1.20 Adenosine kinase**	
		2.7.1.35 Pyridoxal kinase (*)	
		2.7.1.45 2-dehydro-3-deoxygluconokinase	
		2.7.1.49 Hydroxymethylpyrimidine kinase	
		**2.7.1.50 Hydroxyethylthiazole kinase**	
		2.7.1.56 1-phosphofructokinase	
		2.7.1.73 Inosine kinase	
		2.7.1.92 5-dehydro-2-deoxygluconokinase	
		2.7.1.144 Tagatose-6-phosphate kinase	
		2.7.1.146 ADP-dependent phosphofructokinase	
		**2.7.1.147 ADP-dependent glucokinase**	
		2.7.4.7 Phosphomethylpyrimidine kinase (*)	
	
	thiamin pyrophosphokinase *175 sequences*	**2.7.6.2 Thiamin pyrophosphokinase**	PDB: 1ig0
	
	glycerate kinase (previously Group 15) *107 sequences*	2.7.1.31 Glycerate kinase (*)	PDB: 1to6

**Table 2:** Classification of kinase activities by family and fold group, part 2.

*Group*	*Family and PFAM/COG members*	*Kinase Activity (E.C.)*	*Representative PDB or gi*
Group 3: ferredoxin-like fold kinases *10973 sequences*	nucleoside-diphosphate kinase: PF00334 *923 sequences*	**2.7.4.6 Nucleoside-diphosphate kinase**	PDB: 2bef
	
	HPPK: PF01288 *609 sequences*	**2.7.6.3 2-amino-4-hydroxy-6- hydroxymethyldihydropteridine pyrophosphokinase**	PDB: 1eqo
	
	guanido kinases: PF00217 *324 sequences*	2.7.3.1 Guanidoacetate kinase	PDB: 1bg0
		**2.7.3.2 Creatine kinase**	
		**2.7.3.3 Arginine kinase**	
		2.7.3.5 Lombricine kinase	
	
	histidine kinase: PF00512, COG2172 *9117 sequences*	**2.7.1.37 Protein kinase (Histidine kinase)**	PDB: 1i59
		**2.7.1.99 [Pyruvate dehydrogenase(lipoamide)] kinase**	
		**2.7.1.115 [3-methyl-2-oxobutanoate dehydrogenase (lipoamide)] kinase**	

Group 4: ribonuclease H-like *2768 sequences*	COG0837, PF00349, PF00370, PF00871	**2.7.1.1 Hexokinase**	PDB: 1dgk
		2.7.1.2 Glucokinase	
		2.7.1.4 Fructokinase	
		2.7.1.5 Rhamnulokinase	
		2.7.1.7 Mannokinase	
		2.7.1.12 Gluconokinase	
		2.7.1.16 L-ribulokinase	
		2.7.1.17 Xylulokinase	
		2.7.1.27 Erythritol kinase	
		**2.7.1.30 Glycerol kinase **	
		2.7.1.33 Pantothenate kinase	
		2.7.1.47 D-ribulokinase	
		2.7.1.51 L-fuculokinase	
		2.7.1.53 L-xylulokinase	
		2.7.1.55 Allose kinase	
		2.7.1.58 2-dehydro-3-deoxygalactonokinase	
		2.7.1.59 N-acetylglucosamine kinase	
		2.7.1.60 N-acylmannosamine kinase	
		2.7.1.63 Polyphosphate-glucose phosphotransferase	
		2.7.1.85 Beta-glucoside kinase	
		**2.7.2.1 Acetate kinase**	
		2.7.2.7 Butyrate kinase	
		2.7.2.14 Branched-chain-fatty-acid kinase	
		2.7.2.- Propionate kinase	

Group 5: TIM β/α-barrel kinase *1119 sequences*	PF00224	**2.7.1.40 Pyruvate kinase**	PDB: 1a49

Group 6: GHMP kinase *885 sequences*	COG1685, COG1907, PF00288, PF01971	2.7.1.6 Galactokinase (*)	PDB: 1h72
		**2.7.1.36 Mevalonate kinase**	
		**2.7.1.39 Homoserine kinase**	
		2.7.1.46 L-arabinokinase	
		2.7.1.52 Fucokinase	
		2.7.1.71 Shikimate kinase	
		2.7.1.148 4-(cytidine 5'-diphospho)-2-*C-*methyl-D- erythritol kinase (*)	
		**2.7.4.2 Phosphomevalonate kinase**	

Group 7: AIR synthetase-like *1843 sequences*	PF00586	2.7.4.16 Thiamine-phosphate kinase	PDB: 1cli
		2.7.9.3 Selenide, water dikinase	

Group 8: riboflavin kinase (previously Group 10) *565 sequences*	PF01687	2.7.1.26 Riboflavin kinase (*)	PDB: 1nb9

Group 9: dihydroxyacetone kinase (previously Group 17) *197 sequences*		2.7.1.29 Glycerone kinase (*)	PDB: 1un9

Group 10: putative glycerate kinase (previously Group 16) *148 sequences*	COG2379	2.7.1.31 Glycerate kinase (*)	PDB: 1o0u

Group 11: polyphosphate kinase (previously Group 9) *446 sequences*	PF02503	2.7.4.1 Polyphosphate kinase	gi|7465499 [48..730]

Group 12: integral membrane kinases (previously Group 8) *263 sequences*	dolichol kinase: PF01879 *127 sequences*	2.7.1.108 Dolichol kinase	gi|6323655 [349..513]
	
	undecaprenol kinase *136 sequences*	2.7.1.66 Undecaprenol kinase	gi|1705428

Occurrences of the same kinase activity in more than one family reflect cases of convergent evolution to the same kinase activity from different ancestors. For example, homoserine kinase entries are found in the protein kinase-like family (Group 1) and the GHMP kinase family (Group 6). These proteins each carry out the same biochemical reaction and therefore have identical EC specifications, but they belong to two unrelated protein families. Currently, an experimental structure is available only for the homoserine kinase from the GHMP kinase family.

### Framework of the classification remains unchanged

The updated classification includes 42,092 additional sequences, 343 additional kinase structures, and 12 additional kinase specificities compared to the original classification. Although the total number of kinase sequences included in the classification has an impressive increase of more than three-fold (from 17,310 to 59,402), all new kinase sequences were found to be homologous to the previously established families, and thus are contained in the existing family and fold group classification. Furthermore, 343 additional kinase structures have been solved since the initial classification was completed. The majority of these structures correspond to kinases for which at least one representative structure was already known. For example, dozens of additional eukaryotic protein serine-threonine/tyrosine kinase structures were solved. Structures of 20 kinases with previously uncharacterized structures were also published (indicated by asterisks in Tables 1 and 2). The structural folds for 15 of these 20 kinases were predicted by our initial kinase classification based on their homology to proteins with known structures. All 15 of these predicted folds were shown be to correct by the experimentally determined structures. For example, choline kinase was expected to have a protein kinase-like fold similar to the other members of Family 1a (protein S/T-Y kinases and atypical protein kinases). The crystal structure of choline kinase [2] shows that this protein does indeed adopt a eukaryotic protein kinase-like fold. Likewise, pyridoxal kinase was shown to have a ribokinase-like fold [3], as was predicted in the initial kinase classification. Thus, the placements of these kinases in the classification scheme remain unchanged.

The five remaining kinases with recently solved structures belong to families for which the fold was previously unknown. Two of these kinases, riboflavin kinase and dihydroxyacetone kinase, represent two new unique kinase folds. One glycerate kinase family, which was previously listed as an independent fold group, is now placed as an additional family in the Rossmann-like fold group due to similarities in architecture and topology of the predicted nucleotide-binding domain. As the nucleotide-binding domain cannot be confidently predicted for a second distinct glycerate kinase family, these sequences tentatively remain as a separate fold group. Lastly, inositol 1,4,5-trisphosphate 3-kinase is now known to be a member of the lipid kinase-like family (Family 2b).

The total numbers of sequences, structures, families, and fold groups in the initial and updated classifications are summarized in Table 3.

**Table 3:** Comparison of initial and updated kinase surveys.

	***Sequences***	***Structures***	***Families***	***Families with Known Structure***	***Fold Groups***	***Fold Groups with Known Structure***
Initial Survey	17310	359	30	19	17	7
Updated Survey	59402	702	25	22	12	10

### Two new kinase folds are characterized

**Figure 1:**
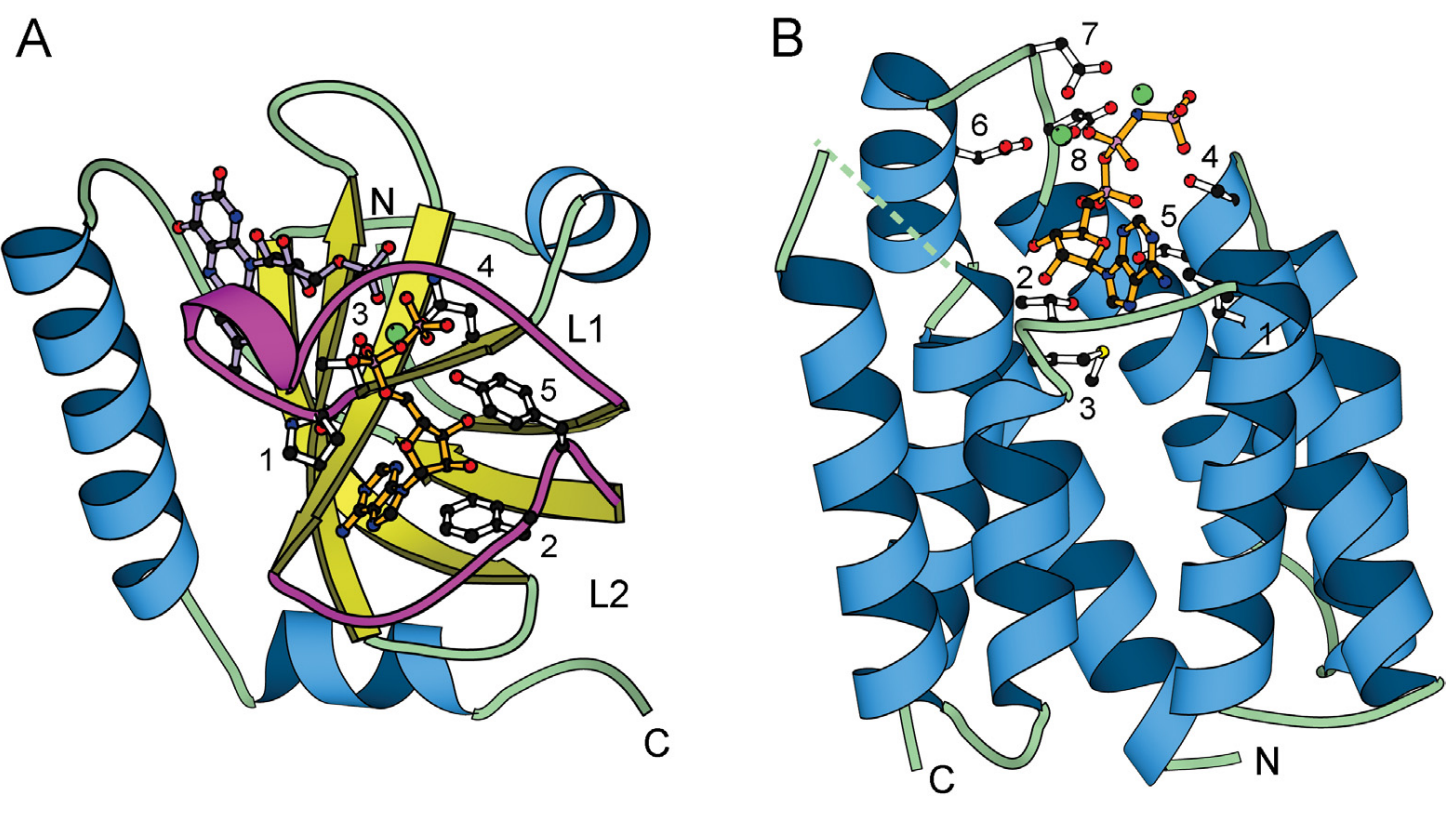
**Two new kinase folds**. a) Riboflavin kinase (PDB|1q9s [8]). Loops L1 and L2 are shown in magenta. Residues **1 **(Pro33) and **2 **(Phe97) interact with the adenine ring of the nucleotide. Residues **3 **(Thr34) and **4 **(Asn36) coordinate the Mg^2+ ^cation. Residues **4 **(Asn36) and **5 **(Tyr98) interact with the phosphate tail of the nucleotide. In this and all other structure figures, the ATP analog is colored orange, substrate molecules are purple, and Mg^2+ ^cations are green balls. Ribbon diagrams were made using the MOLSCRIPT [57] program. b) Dihydroxyacetone kinase nucleotide-binding domain (PDB|1un9 [9]). Residues **1 **(Leu435), **2 **(Thr476), and **3 **(Met477) pack around the adenine ring of the nucleotide. Residues **4 **(Ser431) and **5 **(Ser432) interact with the phosphate tail of the nucleotide. Residues **6 **(Asp380), **7 **(Asp385), and **8 **(Asp387) are involved in coordinating the two Mg^2+ ^cations. Dashed lines indicate disordered regions in the structure.

During the last two years the representative structures of Group 10 (riboflavin kinase) and Group 17 (dihydroxyacetone kinase) in the original kinase classification have been characterized, adding two new folds in the kinase fold repertoire. Riboflavin kinase (RFK) is an essential enzyme in the flavin cofactor biosynthetic pathway in both prokaryotes and eukaryotes [4,5]. The core of the riboflavin kinase structure [6,7] contains a 6-stranded β barrel with Greek key topology (Figure 1a). This is the only kinase currently known to belong to the all-β class of proteins. The bound nucleotide is situated at one end of the β-barrel between two loop regions (L1 and L2), one of which contains a short 3_10 _helix. The solvent-exposed L1 loop and 3_10 _helix form an arch, under which the ADP phosphate tail and requisite Mg^2+ ^cation bind. The adenine ring is positioned by the Pro33 and Phe97 side chains, while the ADP β-phosphate interacts with the hydroxyl group of Tyr98 and the amino group of Asn36. However, the majority of contacts with the nucleotide are made by main chain amide and carbonyl groups from the two loop regions and the 3_10 _helix (shown in magenta in Figure 1a). The tightly bound Mg^2+ ^ion is coordinated directly to the side chains of Thr34 and Asn36, to the α-and β-phosphates of ADP, and presumably to the γ-phosphate of ATP as well [7,8]. Thr34 and Asn36 are part of the unique signature PTAN motif of riboflavin kinases which is located on a short β-strand following loop L1 and the 3_10 _helix. Drastic conformational changes induced by binding of either nucleotide or flavin ligand have been demonstrated [7,8]. Because the fold of riboflavin kinase is not similar to any other known kinase structure, it remains as a separate fold group (now Group 8) in our kinase classification.

The second new kinase fold was revealed by the structure of the ATP-dependent dihydroxyacetone kinase from *Citrobacter freundii *[9]. Dihydroxyacetone kinase sequences are also widely distributed in organisms in all three kingdoms of life. This protein contains two regions separated by an extended linker. The N-terminal region (termed K-domain) is homologous to the non-ATP dependent DhaK protein in *E. coli *and other gram negative bacteria. It consists of two α/β domains and is responsible for dihydroxyacetone binding. The C-terminal region (termed L-domain, homologous to the DhaL protein in *E. coli*) is the nucleotide-binding domain and is comprised of 8 antiparallel α-helices that form a closed barrel (Figure 1b). The α-helices are all slightly tilted away from the axis of the barrel, forming a pocket in which a phospholipid is bound. The bound ATP analog is found to be located at the top of the α-barrel. The N-terminus of one helix (H4) is pointed toward the γ-phosphate of ATP and, together with a glycine-rich loop between helices H3 and H4, forms the primary binding site for the ATP phosphates. Ser432 interacts with the ATP α-phosphate, while Ser431 interacts with the ATP β-and γ-phosphates. Two Mg^2+ ^ions are coordinated by all three phosphates of ATP and by the three highly conserved aspartates (Asp380, Asp385 and Asp387) located on a loop between helices H1 and H2. Additionally, the adenine ring is packed against several hydrophobic side chains (Leu435, Thr476, and Met477). Dihydroxyacetone kinase is the only kinase known to have an all-α nucleotide-binding domain. It represents another new fold group (now Group 10) in our kinase classification scheme as its fold is unlike any other kinase with known structure.

### Two glycerate kinase families now with solved structures

The initial kinase survey included two protein families with putative glycerate kinase activity. These proteins fall into two separate fold groups since no significant sequence similarity was detected between the two families despite their presumably identical biochemical activities. One family (previously Group 15) contains glycerate kinases from bacterial species, primarily of the firmicutes group and of the gamma subdivision of the proteobacterial group. The second family (previously Group 16) consists of proteins from eukaryotes and archaea in addition to several different bacterial species. Representative structures from each of these two families have recently been solved.

**Figure 2:**
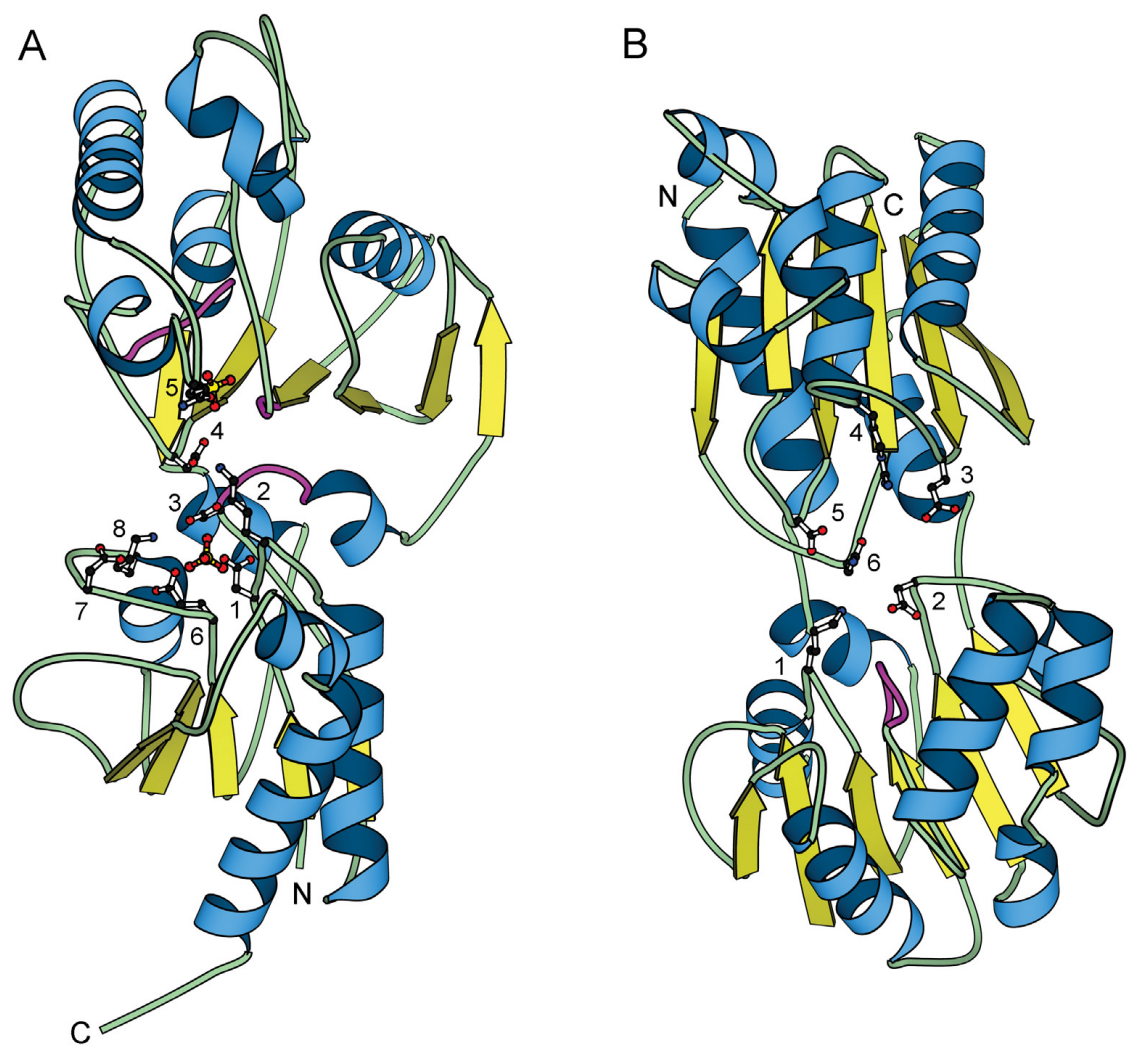
**Glycerate kinase structures**. a) *Neisseria meningitides *glycerate kinase (PDB|1to6), a representative of first glycerate kinase family (previously Group 15). Highly conserved amino acids with side chains pointing into the presumed active site include 6 residues from the Rossmann-like domain (**1 **– Asp8, **2 **– Lys11, **3 **– Asp43, **6 **– Glu286, **7 **– Asp290, and **8 **– Lys297) and 2 residues from the inserted domain (**4 **– Asp191 and **5 **– Gln209). Glycine rich loops are shown in magenta. Sulfates are shown in ball-and-stick representation. b) *Thermotoga maritima *putative glycerate kinase (PDB|1o0u), a member of the second glycerate kinase family (previously Group 16, now Group 10). Highly conserved amino acids with side chains pointing into the presumed active site include 2 residues from the Rossmann-like domain (**1 **– Lys47 and **2 **– Asp189) and 4 residues from the C-terminal domain (**3 **– Glu312, **4 **– Arg325, **5 **– Asp351, and **6 **– Asn407). The glycine rich loop is shown in magenta.

Glycerate kinase from *Neisseria meningitidis *(PDB|1to6) (Rajashankar, K.R., Kniewel, R., Solorzano, V., and Lima, C.D. Glycerate Kinase from *Neisseria meningitidis *(Serogroup A). *To be published*.) is a member of the first glycerate kinase family (previously Group 15). The fold of this protein consists of two non-similar α/β domains (Figure 2a). The N-terminal domain contains a central 5-stranded parallel β-sheet (strand order 21345) and several surrounding helices with Rossmann-like topology. The C-terminal domain contains a central 6-stranded mixed β-sheet (strand order 123456), with strand 2 antiparallel to the rest of the β-sheet. In this structure, the C-terminal domain is inserted between strands 2 and 3 of the N-terminal domain. The active site is likely to be in the cleft between the two α/β domains. Although this structure does not include a bound nucleotide or substrate, the two sulfate groups observed in the presumed active site may indicate the locations of the nucleotide and glycerate binding sites. In this structure, eight highly conserved polar or charged residues have side chains pointing into the presumed active site (Figure 2a). Six of these eight residues, including two lysines, are contributed by the Rossmann-like domain. Furthermore, the crevice in which the sulfate group is located is significantly larger in the Rossmann-like domain that the corresponding crevice in the C-terminal domain, suggesting that the Rossmann-like domain may accommodate a larger molecule such as ATP. Based on the presence of these conserved lysine residues, which are common features of nucleotide binding sites in kinases, and the large crevice that may serve as the ATP-binding site, the Rossmann-like domain is predicted to perform the nucleotide binding role in this kinase. Thus, this family of glycerate kinases is added to the Rossmann-like fold group as a distinct family.

Putative glycerate kinase from *Thermotoga maritima *(PDB|1o0u) (Joint Center for Structural Genomics. Crystal Structure of Glycerate Kinase (TM1585) from *Thermotoga maritima *at 2.95 A Resolution. *To be published*.) is a member of the second glycerate kinase family (previously Group 16). The fold of this protein also consists of two non-similar α/β domains (Figure 2b). The N-terminal α/β domain has Rossmann-like topology with the central 6-stranded β-sheet in the order of 654123. The C-terminal domain contains a 6-stranded mixed β-sheet with strand order 126345 and several helices packed on both sides of the β-sheet. The active site is likely to be in the cleft between the two α/β domains. In this structure, six highly conserved polar or charged residues are found with side chains pointing into the presumed active site (Figure 2b). The C-terminal domain contributes four of these highly conserved residues, while the Rossmann-like domain contributes the remaining two residues in addition to a glycine-rich loop. Each of these domains contains one highly conserved basic residue that could potentially interact with the triphosphate tail of the bound ATP: Lys47 in the Rossmann-like domain and Arg325 in the C-terminal domain. Based on the available information, it is not possible to confidently predict which domain is responsible for nucleotide binding in this putative glycerate kinase. Therefore, this family is kept as a separate fold group until its active site is characterized. The annotation for these putative glycerate kinases is based on a gene found in a 5-kb fragment that is apparently responsible for complementation in *Methylbacterium extorquens *AM1 mutants lacking glycerate kinase activity [10]. However, other family members are annotated as putative glycerate dehydrogenases/hydroxypyruvate reductases based genetic analysis of the tartrate utilization pathway in *Agrobacterium vitis *[11]. Glycerate kinase and glycerate dehydrogenase/hydroxypyruvate reductase catalyze successive steps in the serine metabolism pathway. Therefore, the exact biochemical function of this enzyme family remains to be resolved.

### Inositol 1,4,5-trisphosphate 3-kinase is a member of the lipid kinase-like family

**Figure 3:**
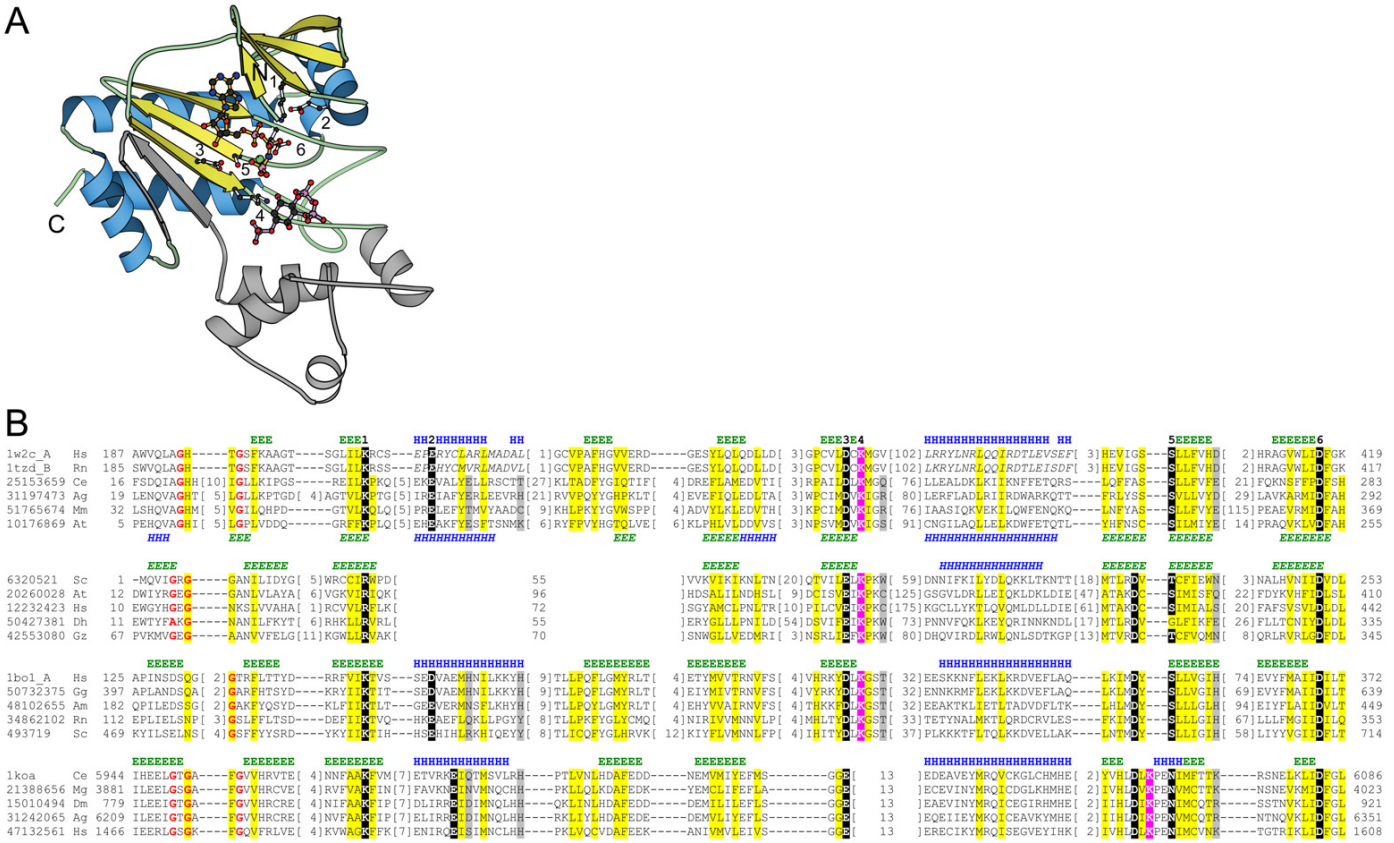
**Structure of inositol polyphosphate kinases**. a) Inositol 1,4,5-trisphosphate 3-kinase (I3P3K) (PDB|1w2c [12]) adopts a lipid kinase/protein kinase-like fold. Common core of lipid kinases, eukaryotic protein kinases, and I3P3K is shown in blue (α-helices) and yellow (β-strands); additional elements are grey. Residue **1 **(Lys209) interacts with the nucleotide's phosphate tail, residue **2 **(Glu215) stabilizes the orientation of residue **1**, residue **3 **(Asp262) binds the sugar group of ATP, residue **4 **(Lys264) likely interacts with the γ-phosphate during transfer, and residues **5 **(Ser399) and **6 **(Asp416) are both likely involved in coordinating two Mg^2+ ^cations. Mn^2+ ^is shown as a green ball and inositol 1,4,5-trisphosphate is shown in purple. b) Multiple sequence alignment of I3P3K (gi|10176869, PDB|1w2c, PDB|1tzd) and I5P2K (gi|6320521) with two related kinase families: lipid kinase representative PIPK (PDB|1bo1 [14]; Family 1b) and protein kinase representative twitchin kinase (PDB|1koa [58]; Family 1a). Italics denote α-helical regions for which the register of structure-based alignment cannot be obtained unequivocally due to significant structural divergence. Critical active site residues are indicated by white bold text highlighted in black/magenta, and are numbered the same as in panel (a). Magenta highlighting indicates residues that perform equivalent roles and are found in equivalent spatial locations, but do not align closely in sequence between the lipid kinase and protein kinase families. In this and other multiple alignments, sequences are labelled according to the NCBI gene identification (gi) number or PDB code and an abbreviation of the species name. Abbreviations in this alignment are as follows: Ag *Anopheles gambiae*, Am *Apis mellifera*, At *Arabidopsis thaliana*, Ce *Caenorhabditis elegans*, Dh *Debaryomyces hansenii*, Dm *Drosophila melanogaster*, Gg *Gallus gallus*, Gz *Gibberella zeae*, Hs *Homo sapiens*, Mg *Mytilus galloprovincialis*, Mm *Mus musculus*, Rn *Rattus norvegicus*, Sc *Saccharomyces cerevisiae*. First and last residue numbers are indicated before and after each sequence. Numbers of excluded residues are specified in square brackets. Residue conservation is denoted with the following scheme: mostly hydrophobic positions, highlighted yellow; mostly charged/polar positions, highlighted grey; small residues, red bold text. Locations of predicted (gi|6320521, gi|10176869) and observed (PDB|1w2c_A, PDB|1tzd_B, PDB|1bo1_A, PDB|1koa) secondary structure elements (E, β-strand; H, α-helix) are marked above the sequences (with the exception of gi|10176869 which is shown below the sequence) in italics and normal font, respectively.

Inositol 1,4,5-trisphosphate 3-kinase (I3P3K) catalyzes the first of several phosphotransfer reactions that convert inositol 1,4,5-trisphosphate, a ubiquitous second messenger in eukaryotes, to inositol pentakisphosphates, inositol hexakisphosphates, and inositol pyrophosphates. I3P3K is a known homolog of other inositol polyphosphate kinases, including inositol 1,4,5-trisphosphate 6-kinase and inositol hexakisphosphate kinase, but does not share significant sequence similarity to any kinase family with a previously solved structure. The solved structures of human I3P3K (PDB|1w2c [12]) and rat I3P3K (PDB|1tzd [13]) reveal that the catalytic core of this kinase adopts a protein kinase-like fold and is comprised of two domains with the active site cleft in between (Figure 3a). The overall structure of I3P3K is most similar to the lipid kinases (PDB|1bo1 [14]), which belong to the same fold group as eukaryotic protein kinases in our kinase classification scheme. The shared structural core between lipid kinases and protein kinases includes all elements of the I3P3K N-terminal domain, and part of the β-sheet (strands 1, 4, and 5) and the three α-helices of the C-terminal domain (Figure 3a).

The mode of nucleotide binding in I3P3K is also very similar to that of eukaryotic protein kinases, as each of the critical nucleotide binding and Mg^2+ ^binding residues in I3P3K plays the same role as a corresponding protein kinase residue. Lys209 (human I3P3K; hI3P3K) forms a hydrogen bond with the α-and β-phosphates of ATP and corresponds to the highly conserved Lys72 in protein kinase A (PKA). This lysine residue is oriented by Glu215 in hI3P3K (corresponding to Glu91 in PKA). A second highly conserved lysine residue (Lys264 in hI3P3K) interacts with the 3-OH phosphate acceptor group of the inositol 1,4,5-trisphosphate substrate and likely stabilizes the γ-phosphate during transfer, similar to the role of Lys168 in PKA. Although Lys264 (hI3P3K) and Lys168 (PKA) are contributed by different structural elements in different regions of the protein sequence, these residues are found in equivalent spatial locations and likely play the same role in catalysis. A Mn^2+ ^ion is coordinated by Asp416, which corresponds to the conserved magnesium-binding residue Asp184 of the DFG motif in PKA. Ser399 is expected to coordinate a second divalent cation that is not modeled in the I3P3K structure, as this residue is found in the equivalent spatial location of the conserved magnesium-binding residue Asn171 in PKA. These active site similarities also extend to members of the lipid kinase family, such as phosphatidylinositol phosphate kinase IIβ (PIPK; PDB|1bo1) (Figure 3b), although a representative structure with bound nucleotide has not yet been solved.

Although I3P3K shares similarity of the overall fold as well as the active site with the related lipid kinase and protein kinase-like families, I3P3K is more closely related to the lipid kinase family. I3P3K and the lipid kinases share conserved motifs, including the substrate-binding/catalytic "DLK" motif (Asp262 to Lys264 in hI3P3K) and the magnesium-binding "SSLL" motif (Ser398 to Leu401 in hI3P3K), which are not found in protein kinases. Additionally, DALI [15] identifies lipid kinase representative PIPK (PDB|1bo1) as the closest structural neighbor of I3P3K [13]. Thus, based on the similarity of structural fold and the conservation of critical nucleotide-binding, magnesium-binding, and catalytic residues I3P3K can be assigned to the lipid kinase family in the kinase classification (Family 1b) despite the lack of significant sequence similarity.

### Predicted folds for remaining kinases with unknown structures

Fold predictions (see Methods) were made for each remaining family of kinases without a solved structure, with the exception of the integral membrane kinases (dolichol kinase and undecaprenol kinase). These kinases include inositol 1,4,5-trisphosphate 3-kinase, inositol 1,3,4,5,6-pentakisphosphate 2-kinase, eukaryotic pantothenate kinase, and polyphosphate kinase.

Inositol 1,4,5-trisphosphate 3-kinase (I3P3K; previously Group 11) and inositol 1,3,4,5,6-pentakisphosphate 2-kinase (I5P2K; previously Group 12) both catalyze phosphorylation reactions in the production of inositol polyphosphate (IP) second messengers. These kinases were placed in separate fold groups in the initial survey based on a lack of significant sequence similarity to each other or any other known kinase family. Before the crystal structures of I3P3K were reported during the preparation of this manuscript, fold predictions for both I3P3K and I5P2P were carried out. The results of fold predictions guided by 3D-Jury [16], secondary structure predictions, and observed presence of critical conserved sequence motifs indicated that both of these IP kinases would likely adopt a structural fold similar to lipid kinases and eukaryotic protein kinases, which are possibly related families that share a common ATP-binding site and structural core [17]. Furthermore, a multiple alignment of representative I3P3K, I5P2K, lipid kinase, and protein kinase sequences shows that the critical functional residues in these proteins are also conserved in the IP kinases (Figure 3b). For example, I3P3K and I5P2K each have the conserved lysine/arginine residue that is important for orienting the α-and β-phosphates of nucleotide's triphosphate tail and the aspartate/glutamate that interacts with the ribose of ATP (residues **1 **and **3 **in Figure 3b, respectively). Additionally, the serine/threonine and the aspartate residues involved in coordinating the two requisite Mg^2+ ^cations are conserved in these kinases as well (residues **5 **and **6 **in Figure 3b, respectively). Both I3P3K and I5P2K also have a predicted glycine-rich loop in the N-terminal region of the protein. From the multiple sequence alignment, it becomes apparent that I3P3K and I5P2K are more closely related to the other lipid kinases than to protein kinases. In addition to phosphorylating similar substrates, the IP kinases and lipid kinase family members each have critical active site lysine residue involved in stabilizing the γ-phosphate of ATP during transfer (residue **4 **in Figure 3b) that has migrated in the sequence/structure relative to the protein kinase-like family. The solved structures of I3P3K from human [12] and rat [13], which were published during manuscript preparation and are discussed above, confirm this non-trivial fold assignment as well as the predicted functional roles played by the conserved active site residues. This further increased our confidence in the I5P2K prediction. Thus, I3P3K and I5P2K are now assigned as members of the lipid kinase-like family (Family 1b) in the kinase classification.

**Figure 4:**
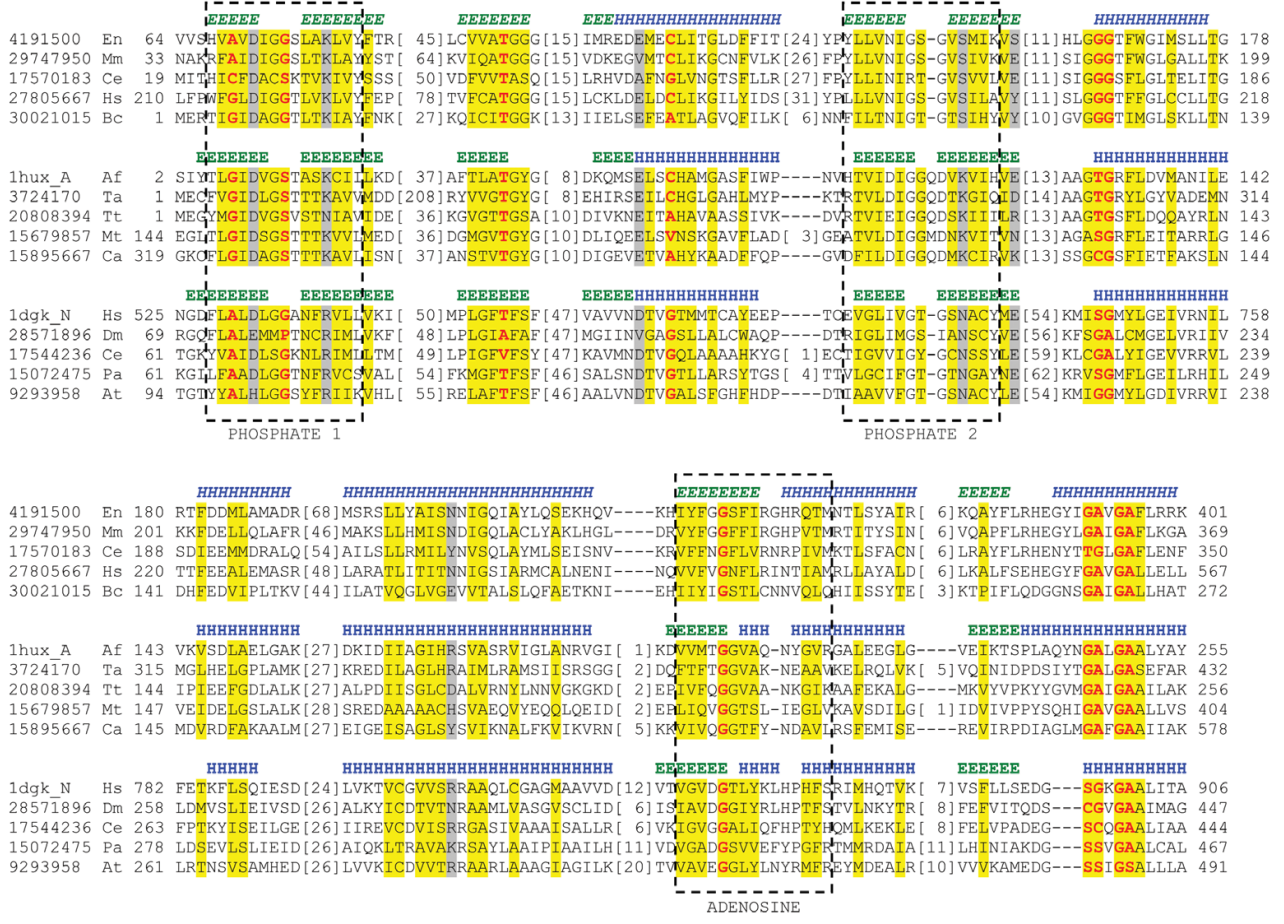
**Eukaryotic pantothenate kinase is a ribonuclease H-like kinase**. Multiple sequence alignment for representative sequences of the pantothenate kinase family and two related ribonuclease H-like families with known structure (2-hydroxyglutaryl-CoA dehydratase component A (PDB|1hux [20]) and hexokinase I (PDB|1dgk [59])) is shown. The PHOSPHATE 1, PHOSPHATE 2, and ADENOSINE motifs are indicated by dashed boxes. Abbreviations of species names are as follows: Af *Acidaminococcus fermentans*, At *Arabidopsis thaliana*, Bc *Bacillus cereus*, Ca *Clostridium acetobutylicum*, Ce *Caenorhabditis elegans*, Dm *Drosophila melanogaster*, En *Emericella nidulans*, Hs *Homo sapiens*, Mm *Mus musculus*, Mt *Methanothermobacter thermautotrophicus*, Pa *Pichia angusta*, Ta *Thauera aromatica*, Tt *Thermoanaerobacter tengcongensis*. Locations of predicted (gi|4191500) and observed (PDB|1hux_A, PDB|1dgk_N) secondary structure elements (E, β-strand; H, α-helix) are marked above the sequences in italics and normal font, respectively.

The spatial structure of eukaryotic pantothenate kinase (previously Group 14) is unknown. The crystal structure of prokaryotic pantothenate kinase identifies this enzyme as a member of the P-loop kinase family [18]. However, due to the lack of sequence identity between the prokaryotic and eukaryotic versions of this protein [19] in conjunction with dissimilar predicted secondary structure patterns, eukaryotic pantothenate kinase is expected to adopt a fold distinct from its prokaryotic counterpart. Although standard sequence similarity search methods failed to obtain any reasonable structural assignment, several fold recognition servers strongly suggested that the eukaryotic pantothenate kinases adopt a ribonuclease H-like fold. The ribonuclease H-like fold is composed of three layers (α/β/α), including a 5-stranded mixed β-sheet with strand order 32145, where strand 2 antiparallel to the rest of the sheet. The topology of the core of this fold is βββαβαβα. A duplication of this fold is also seen in several other ribonuclease H-like kinases (Group 4), although the closest structural template identified by 3D-Jury is a non-kinase homolog of this family (2-hydroxyglutaryl-CoA dehydratase component A; PDB|1hux [20]). Importantly, the ribonuclease H-like kinase group contains the ASKHA (acetate and sugar kinase/hsp70/actin) superfamily. Nucleotide binding and divalent metal coordination are achieved by several motifs conserved within the ASKHA superfamily [21]. These conserved motifs include the ADENOSINE motif that interacts with the ribosyl and the α-phosphoryl group of ATP, the PHOSPHATE 1 motif that interacts with Mg^2+ ^through coordinated water molecules, and the PHOSPHATE 2 motif that interacts with the β-and γ-phosphoryl groups of ATP. The conservation of these motifs in the eukaryotic pantothenate kinases is noted in Figure 4. Thus, based on the presence of these distinguishing motifs in addition to the similarity of secondary structure patterns (Figure 4), the eukaryotic pantothenate kinases are added to the ribonuclease H-like family.

**Figure 5:**
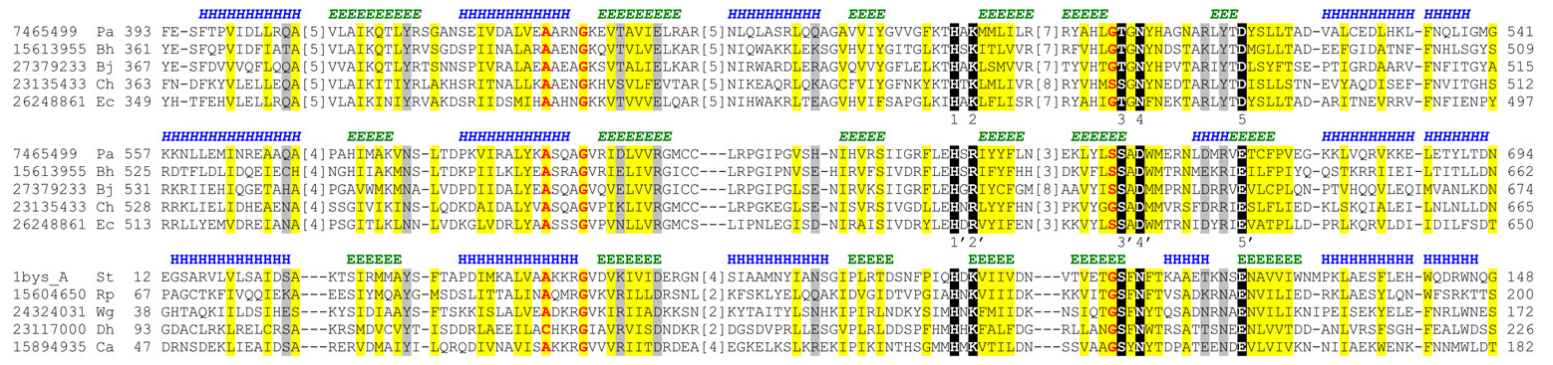
**Second and third domains of polyphosphate kinase are homologous to phospholipase D**. Multiple sequence alignment for representative sequences of polyphosphate kinase (gi|7465499, Group 11) and the phospholipase D family (PDB|1bys [24]) is shown. Highly conserved active site residues are highlighted in black and shown in white bold text. Abbreviations of species names are as follows: Bh *Bacillus halodurans*, Bj *Bradyrhizobium japonicum*, Ca *Clostridium acetobutylicum*, Ch *Cytophaga hutchinsonii*, Dh *Desulfitobacterium hafniense*, Ec *Escherichia coli*, Pa *Pseudomonas aeruginosa*, Rp *Rickettsia prowazekii*, St *Salmonella typhimurium*, Wg *Wigglesworthia glossinidia brevipalpis*. Locations of predicted (gi|7465499) and observed (PDB|1bys_A) secondary structure elements (E, β-strand; H, α-helix) are marked above the sequences in italics and normal font, respectively.

Polyphosphate kinase (PPK) synthesizes inorganic polyphosphate (polyP) by catalyzing the transfer of the γ-phosphate of ATP to a linear polymer of tens or hundreds of orthophosphate residues. Additionally, this enzyme can catalyze the transfer of a phosphate group from polyP to ADP or GDP and can generate ppppG by transferring a pyrophosphate group from polyP to GDP as well [22]. PPK sequences have been identified in many bacterial species as well as in some archaea. Fold predictions indicate that PPK is comprised of three domains: two phospholipase D-like subdomains, which are clearly recognizable by standard sequence comparison methods such as RPS-BLAST, and an N-terminal Rossmann-like domain. The phospholipase D (PLD) fold consists of a 7-stranded mixed β-sheet (strand order 1765234) flanked by several α-helices. PPK contains two PLD-like subdomains, presumably arranged in the same manner as other PLD superfamily members such as tyrosyl-DNA phosphodiesterase (PDB|1jy1 [23]) and the homodimer of bacterial endonuclease Nuc from *Salmonella typhimurium *(PDB|1bys [24]), which is the closest structural template identified by 3D-Jury [16]. The active site of enzymes in the PLD superfamily is located between two PLD-like subdomains. This active site is highly symmetrical due to the five equivalent highly conserved residues that are contributed by each PLD-like subdomain. Among these are two histidine residues (one from each subdomain) proposed to perform critical catalytic roles. One histidine may act as a nucleophile by attacking the phosphodiester bond that is cleaved by known PLD-like enzymes and form a phospho-histidine intermediate, while the second conserved histidine could serve as a general acid by protonating the leaving group [24]. The conservation of these critical active site residues is shown in Figure 5. Consistent with the proposed mechanism for PLD-like enzymes, PPK has been shown to form a phospho-histidine intermediate during the phosphotransfer reaction [25,26] (residue **1 **in Figure 5). If nucleotide binding in the kinase reaction is carried out by the PLD-like domains, PPK will denote a new fold group in the kinase classification since the PLD-like topology is unlike any exiting kinase fold group. However, the possibility that N-terminal domain of PPK may be responsible for binding of ATP cannot be ruled out at this point. Fold prediction suggests that the PPK N-terminal region likely adopts a Rossmann-like fold, which is also seen in thousands of other kinase sequences (Group 2). We are currently unable to confidently predict whether the Rossmann-like or PLD-like region of PPK is responsible for nucleotide binding in the kinase reaction. Thus, PPK is presently considered a separate fold group in our kinase classification.

### Comprehensive structural annotation of kinases

Of the 25 kinase families, 22 currently have at least one homolog with a solved structure (Tables 1 and 2). The structural folds of each domain within one additional family (polyphosphate kinase) are predicted as discussed above. The two remaining families are integral membrane kinases. Although the tertiary structure of dolichol kinase and undecaprenol kinase are not yet determined, secondary structure predictions indicate that both of these families adopt all α-helical conformations. Thus, structural annotations of all kinase families are now revealed, including fold descriptions for all globular kinases, and the kinase fold groups listed in Tables 1 and 2 present the complete structural depiction of this entire functional class of proteins. The structural folds adopted by kinases include some of the most widely spread protein folds, including the Rossmann-like fold, ferredoxin-like fold, and TIM β/α-barrel fold. The kinase fold repertoire also includes representatives of all major classes (all α, all β, α+β, α/β) of protein structures, demonstrating that nature has found ways to utilize all varieties of secondary structure combinations to carry out the kinase reaction.

### Additional kinase activities in the classification

The updated classification also includes 12 additional kinase activities. However, 7 of these activities reflect changes within the EC database rather than newly characterized kinase sequences. For example, while the structure of adenosylcobinamide kinase was published in 1998, its EC number (EC 2.7.1.156) was only assigned in April 2004. The sequences and structures of this kinase were included in initial kinase survey (e.g. PDB|1cbu [27]) and were placed in the P-loop kinase family of the Rossmann-like fold group.

The updated kinase classification does include 5 newly annotated or characterized kinases (indicated by underlining in Tables 1 and 2). The first sequences associated with each of these activities (2.7.1.52 Fucokinase, 2.7.1.92 5-dehydro-2-deoxygluconokinase, 2.7.1.138 Ceramide kinase, and 2.7.1.140 Inositol-tetrakisphosphate 5-kinase) were identified after the initial kinase survey was completed. Sequences with [Hydroxymethylglutaryl-CoA reductase (NADPH_2_)] kinase activity (2.7.1.109) were included in the initial classification, although only a general kinase activity ("AMP-activated protein kinase") was assigned at the time. Thus, the specific kinase activity of this enzyme is a new addition to the kinase classification as well.

As can be seen from Tables 1 and 2, all of these newly annotated kinases (underlined in the tables) belong to existing kinase families containing members that are well characterized both biochemically and structurally. The link between these kinases and members of the existing families can all be identified by BLAST [28] with E-values less than 1e^-5^. Therefore, the catalytic mechanisms of these newly annotated kinases may be inferred from their closely related homologs.

### Two families previously annotated as kinases are removed from the classification

Two kinase families were removed from the classification. In both cases, the sequences were annotated as kinases in the NCBI database, but further biochemical studies have indicated that they most likely do not have kinase activity. The case of the ThrH protein (previously Family 2g – L-2-Haloacid dehalogenase-like family) is an unusual one. Earlier genetic studies of the *thrH *gene of *Pseudomonas aeruginosa *have shown that over-expression of ThrH complements both homoserine kinase and phosphoserine phosphatase activities *in vivo*. The gene product of *thrH *was thus annotated as "bifunctional homoserine kinase/phosphoserine phosphatase isoenzyme" [29]. A more recent structural and biochemical study of ThrH has shown that this protein does not have ATP-dependent kinase activity. Instead it possesses phosphoserine phosphatase activity and is also able to transfer a phosphate group from phosphoserine to homoserine, presumably via a phospho-enzyme intermediate [30]. Thus, although ThrH is able to generate phosphohomoserine and complement homoserine kinase activity *in vivo*, it achieves this through a completely different chemical mechanism from that of true homoserine kinase. Thus, ThrH is in fact a phosphatase and phosphotransferase but not a kinase and is subsequently removed from the kinase classification.

The putative tagatose 6-phosphate kinase (T6P kinase, previously Group 13) activity was initially suggested for the *agaZ *gene product based on the computational analysis and reconstruction of the putative N-acetylgalactosamine metabolic pathway in *E. coli *[31]. However, no tagatose 6-phosphate kinase activity for either AgaZ or its homolog GatZ can be detected experimentally, and genetic studies have suggested that *gatZ *is associated with tagatose-1,6-bisphosphate aldolase activity [32,33]. Results of transitive PSI-BLAST searches and fold predictions with 3D-Jury [16] also suggest similarity between AgaZ and tagatose- and fructose-bisphosphate aldolases with TIM β/α barrel fold. The alignment of the putative T6P kinases with the aldolase families revealed that several residues in the aldolases that are involved in substrate binding and catalysis are also conserved in the AgaZ/GatZ protein family (data not shown). These include two histidine residues involved in the coordination of the catalytic Zn^2+ ^and the aspartate residue that is proposed to protonate the substrate during the aldolase reaction [34]. Thus, based on both functional study and structural prediction, it is likely that these proteins carry out an aldolase reaction rather than a kinase reaction. Therefore, this family is removed in the updated kinase classification as well.

### More diversity of structural folds and nucleotide binding in kinases

In the original kinase survey study, the substrate binding and phosphoryl transfer reaction mechanisms were analyzed across protein families and fold groups, and several distinct modes of nucleotide binding have emerged. One recurring theme observed was the bound nucleotide located at the C-terminus of β-strands and N-terminus of α-helices (i.e. on β-α loops). Signature motifs that interact with the nucleotide are also common. These motifs are often rich in glycines and found on both β-α and β-β loops. One such example is the so-called P-loop (phosphate-binding loop) formed by the Walker-A motif, which is found in a variety of different proteins that bind nucleotides [35]. The P-loop, which is located on a β-α loop, wraps around the triphosphate tail of the bound nucleotide. Together with the positive dipole of the α-helix and some positively charged side chains, a strong anion hole is created for the binding of the phosphate groups of the nucleotide.

The newly characterized riboflavin kinase is the only known kinase with an all-β structural core. RFK contains a novel nucleotide-binding motif that encompasses an arched loop (L1 in Fig. 1a), a 3_10 _helix, and a reverse turn followed by a short β-strand (Figure 1a). This short β-strand encompasses the highly conserved PTAN sequence motif. The threonine and asparagine in the motif are directly involved in the coordination of Mg^2+ ^and thus the binding of MgATP (Figure 1a). The mechanism of the phosphotransfer reaction in RFK appears to be direct in-line transfer of the γ-phosphate of ATP to the 5' hydroxyl group of riboflavin, which may be activated by a glutamate residue (Glu86) [7,8]. The most unique features of RFK appear to be that the phosphate is transferred through a hole beneath the highly dynamic Loop L1, and the proper positioning of the catalytic residues depends on binding of the substrates.

The second kinase with a novel fold is dihydroxyacetone kinase, which is the only known kinase with an all-α nucleotide-binding domain. The binding of MgATP is accomplished in part through interactions with a glycine-rich loop between helices H3 and H4 and the N-terminal positive dipole of Helix H4. Uniquely, two Mg^2+ ^ions were found to ligand to the ATP phosphates and a cluster of three highly conserved aspartate residues on a loop between helices H1 and H2. The mechanism of phosphotransfer in DhaK is not clear since the complex conformation of the crystal structure is influenced by the crystal packing and appears not in its active form. A reaction mechanism involving a phospho-enzyme intermediate cannot be ruled out at this point.

Thus, the newly characterized riboflavin kinase and dihydroxyacetone kinase reveal spectacularly different structures compared to those previously known and have enriched the kinase fold repertoire, which now includes all major classes of protein structures with α/β, α+β, all-β, and all-α folds. Although the substrate recognition and catalytic mechanisms of the two newly characterized enzymes share certain features with other kinases, such as utilization of a helix dipole and backbone amide groups in a glycine-rich loop for nucleotide binding, as a whole they are distinctly unique.

### Supplementary material

Supplementary material is available by anonymous ftp at , which includes lists of NCBI gene identification (gi) numbers for sequences from each kinase family and a table cataloging functional residues from kinase family representatives.

## Conclusion

We have performed an updated comprehensive survey of all available kinase sequences and structures. All experimentally characterized kinase families, with the exception of the integral membrane kinases, are now associated with a known or predicted structural fold. Therefore, the kinases are the first large functional class with a comprehensive structural annotation for its known members. Additionally, we find that, despite a three-fold increase in the number of kinase sequences and two-fold increase in the number of kinase structures, the framework of our classification remains sufficient for describing all available kinases. Furthermore, we find that no fold predictions made in the initial kinase survey are now shown to be incorrect. Thus, the updated kinase survey serves to confirm the soundness of our classification scheme in addition to presenting the final global picture of this entire functional class. Potential uses for this classification include deduction of protein function, structural fold, or enzymatic mechanism of poorly studied or newly discovered kinases based on proteins in the same family.

## Methods

### Constructing updated families of homologous kinases sequences

The updated version of the kinase classification scheme was assembled with the same strategy that was applied in the construction of our first kinase survey [1], with the previous classification used as a framework for this update. Briefly, the hmmsearch program of the HMMER2 package [36] was used to assign sequences from the NCBI non-redundant (nr) database (July 2, 2004; 2,911,742 sequences, including environmental sequences) to a set of 57 profiles describing catalytic kinase domains (E-value cutoff 0.1). This set of kinase profiles (from Pfam [37] version 5.4 and COG version 2 [38]) was constructed during the initial kinase survey. As these profiles had been assembled into families of homologous sequences in the initial classification scheme, the sequences assigned to these profiles by hmmsearch were then placed in the appropriate kinase families. Additionally, the GREFD program of the SEALS package [39] was used to extract from the nr all sequences for which the definition line contained the pattern "kinase". For any kinase sequence not already assigned to a kinase family (either by hmmsearch or in the previous classification), three iterations of PSI-BLAST [28] were carried out against the nr database (E-value cutoff 0.001). Any kinase producing a hit to a sequence already assigned (either by hmmsearch or in the previous classification) was subsequently placed in the corresponding kinase family. The appropriate placement of the remaining unassigned kinase sequences was determined by manual inspection of multiple alignments, secondary structure predictions, and distant PSI-BLAST hits. These proteins were placed into existing kinase families based on the presence of conserved catalytic residues and other distinguishing motifs as well as overall sequence similarity. Sequences that were fragments, non-kinase entries (e.g. kinase inhibitors), or non-catalytic entries (e.g. regulatory subunits) were removed. Such sequences were identified by their annotations in the non-redundant database and by their lengths being too short to cover the complete protein. In the case of non-kinase or non-catalytic entries, lack of kinase activity was confirmed based on either literature available concerning the sequences in question or on obvious homology to a protein with known non-kinase function. The lists of newly identified kinase sequences were appended to those for each of the kinase families included in the initial classification.

The meaning of families and fold groups in the new version of the classification remain unaltered: the families contain homologous kinase sequences, while the fold groups imply similarity of structural fold but not homology.

### Fold group classification

In the initial classification, fold groups were assembled based solely on similarity of structures. Families in the same fold group share structurally similar nucleotide-binding domains that are of the same architecture and topology (or related by circular permutation) for at least the core of the domain. Some of the recently solved kinase structures allowed for the merging of certain kinase families to previously established fold groups based on these same structural similarity guidelines.

### Fold predictions

To provide fold assignments for the remaining structurally uncharacterized kinase families, initial analysis was performed with standard sequence similarity search methods such as transitive PSI-BLAST [28], RPS-BLAST [40], and profile HMMs from SMART [41]. All searches were initiated with the representative sequences (selected in the initial survey [1]) of the families. Transitive PSI-BLAST (E-value threshold 0.01) was run against the NCBI non-redundant protein sequence database until convergence. CDD (RPS-BLAST) [42] and SMART (profile HMMs) [43] web tools were used with default settings to detect distant homology to other conserved protein domains annotated in the SMART, PFAM [37] and COG [44] databases. In addition, RPS-BLAST was also exploited to compare query sequences directly to the PDB using the GRDB system [45]. Further analysis was carried out using Meta Server [46], which assembles various secondary structure prediction and fold recognition methods. Collected predictions were screened with 3D-Jury [16], the consensus method of fold recognition servers. The default servers used by the 3D-Jury system for consensus building include: ORFeus [47], Meta-BASIC [48]., FFAS03 [49], mGenTHREADER [50], INBGU [51], RAPTOR [52], FUGUE-2 [53], and 3D-PSSM [54]. Final fold/template selections were based on 3D-Jury reliability scores as well as those of individual servers, correctness of mapping of predicted and observed secondary structure elements, and conservation of functionally and/or structurally important residues. In the case of inositol 1,3,4,5,6-pentakisphosphate 2-kinase, initial fold assignment was based on functional analogy to 1-phosphatidylinositol-4-phoshate 5-kinase, which phosphorylates similar substrates.

### Sequence-to-structure alignments

Multiple sequence alignments for considered protein families were prepared using PCMA [55] followed by manual adjustment. Sequence-to-structure alignments between analyzed kinase families and their distantly related template families were built using consensus alignment approach and 3D assessment [56] based mainly on 3D-Jury results for representative kinase sequences. Sequences of distantly related proteins of known structure were aligned first based on the superposition of their 3D structures. In the case of inositol 1,3,4,5,6-pentakisphosphate 2-kinase, sequence-to-structure alignment was prepared manually with respect to the results of secondary structure predictions and the preservation of functionally critical residues as well as the hydrophobic core of the protein.

### Alterations within the classification

Although the framework of the classification remains essentially unchanged, the organization within the classification has been slightly modified. More specifically, the numbering of the fold groups has been adjusted so that all kinase families with unsolved structures are at the end. Furthermore, the EC (Enzyme Commission) numbers have been updated to reflect the current organization of the EC database. Therefore, the EC content of each family may differ somewhat between the initial and updated classifications, but these changes do not indicate new additions to the family unless otherwise indicated.

## Authors' contributions

SC performed the update of the classification and participated in drafting the manuscript. KG performed fold predictions. HZ participated in drafting the manuscript. NVG conceived of the study and participated in its design and coordination. All authors read and approved the final manuscript.
